# Exchange Transfusion for Neonatal Hyperbilirubinemia in Johannesburg, South Africa, from 2006 to 2011

**DOI:** 10.1155/2016/1268149

**Published:** 2016-02-29

**Authors:** Daynia E. Ballot, Gilbert Rugamba

**Affiliations:** Department of Paediatrics and Child Health, University of the Witwatersrand, Private Bag Box 39, Johannesburg 2000, South Africa

## Abstract

*Background*. Severe hyperbilirubinaemia requiring exchange transfusion has become less common in recent years; however, kernicterus still occurs. The aim of this study was to review babies undergoing exchange transfusion for severe hyperbilirubinaemia in a Johannesburg hospital.* Methodology*. This was a retrospective review of babies who required exchange transfusion in both the neonatal and the paediatric wards from June 1, 2006, to December 31, 2011.* Results*. There were 64 patients who underwent 67 exchange transfusions. Isoimmune haemolysis (both Rh and ABO incompatibility) was the cause of jaundice in 9/64 (14%). Most babies who underwent exchange transfusion were sick or preterm and were admitted in hospital after birth (38/64; 59.5%); three of these babies died, but not during the exchange transfusion (3/38; 7.9%); all three had signs suggestive of neonatal sepsis. The remaining 26 babies (40.6%) were readmitted to the paediatric wards for exchange transfusion. Six of these babies (6/26; 23.0%) had signs of kernicterus. The most significant complication of exchange transfusion was apnoea requiring mechanical ventilation in three patients (3/64; 4.6%).* Conclusion*. Despite a relatively low number of babies undergoing exchange transfusion, kernicterus still occurs and must be prevented. Proper protocols for screening and management of severe hyperbilirubinaemia need to be enforced.

## 1. Background

Almost all newborn infants develop some degree of hyperbilirubinemia as a normal transition in physiology. High levels of unbound unconjugated bilirubin can cross the blood brain barrier and cause neurological symptoms. The association between hyperbilirubinemia and encephalopathy was first described in 1847 by Hervieux; the term kernicterus was first used by Schmorl in 1903 to describe yellow staining of the basal ganglia [1]. The terms “Bilirubin Induced Neurological Dysfunction” (BIND) and kernicterus are often used interchangeably, although some authors consider BIND to refer to the clinical presentation and kernicterus to be an anatomical diagnosis [2]. In this paper, the term kernicterus will be used to describe both the neuropathology of bilirubin induced brain injury and the associated clinical presentation.

Kernicterus is a preventable condition; the unconjugated bilirubin levels can be decreased to safe levels by the use of phototherapy or exchange transfusion. Phototherapy is an effective way of decreasing the bilirubin level in neonates, by converting unconjugated bilirubin into isomers such as lumirubin that are water soluble and can be excreted in the urine [3]. Exchange transfusion (ET), however, is considered to be the most effective and quickest method to lower the bilirubin level in infants at high risk of kernicterus [4]. An ET is indicated when hyperbilirubinemia remains at dangerous levels despite intensive phototherapy and is particularly useful when there is excessive haemolysis [5]. The actual bilirubin level at which to implement phototherapy or ET has been a subject of controversy for many years. In South Africa, consensus was reached in 2006 on a nationally accepted nomogram for the management of hyperbilirubinemia [6].

Kernicterus is a preventable condition; however cases still occur, even in high-income countries like the USA. Reasons include early discharge of neonates without predischarge screening, lack of knowledge regarding neonatal hyperbilirubinemia among both health workers and caregivers, inadequate follow-up, and an increase in breastfeeding rates without adequate support [7]. Kernicterus and severe hyperbilirubinemia are even more prevalent in low- and middle-income countries, due to delays, at all levels of care, in babies with severe hyperbilirubinemia receiving adequate treatment [8]. Although ET is effective in preventing kernicterus, the procedure has significant complications, including cardiovascular, biochemical, and haematological abnormalities; the risk of death may be as high as 5% [9].

The aim of this study was to review neonates undergoing ET at Charlotte Maxeke Johannesburg Academic Hospital (CMJAH.) Objectives included describing the characteristics of babies undergoing ET, comparing readmitted babies with babies who were inpatients after birth, and describing complications and outcome of ET.

## 2. Subjects and Methods

This was a retrospective review of all newborns undergoing ET for severe hyperbilirubinemia at CMJAH between 01 June 2006 and 30 December 2011. Babies who underwent ET included those who were discharged at birth and readmitted to the general paediatric wards (“readmitted babies”) and those who were still inpatients after birth (“in-hospital babies”). Subjects were identified from the CMJAH neonatal database, admission registers for the paediatric wards, and the South African National Blood Bank database. Subjects with no retrievable records were excluded from the study.

Neonates were managed according to the South African consensus guidelines [6] at the discretion of the attending paediatrician. Phototherapy was commenced in all neonates with severe hyperbilirubinemia; ET was done if the bilirubin remained above the ET levels in the consensus nomogram. Ethics approval was obtained from the Human Research Ethics Committee of the University of the Witwatersrand.

### 2.1. Statistical Methods

Results were described using measures of central tendency, mean and standard deviation for continuous data with a normal distribution or median and range for skewed data. Categorical variables were described as frequency and percentage. Comparisons between continuous variables were done using paired *t*-tests for data with a normal distribution and Wilcoxon signed rank test was used to compare skewed data. A *p* value <0.05 was considered statistically significant. Statistical analysis was done using IBM SPSS version 22.

## 3. Results

A total of 69 babies underwent ET at CMJAH during the study period. Records could not be traced in 5 babies, so there were 64 babies included in the analysis who underwent 67 ET. Demographic and clinical characteristics are shown in Table 1. Most ET (43/67, 64.1%) were done after 72 hours of age. The median age of discharge after ET was 8 days (range 3 to 71).

“Readmitted” and “in-hospital” babies are compared in Table 2. In comparison to the “in-hospital” babies, “readmitted” babies were of significantly greater birth weight and gestational age, had a higher initial total bilirubin, were more likely to be breastfed, and had ET at a later stage (see Table 2). In most cases the cause of neonatal hyperbilirubinemia was undetermined (19/26 (73%) of readmitted babies compared to 32/38 (84%) in “in-hospital” babies (*p* = 0.17)).

### 3.1. Readmitted Babies

A total of 26/64 (40.6%) babies were admitted to the general paediatric wards for ET. Nine of these (34.6%) were transferred from other hospitals. Six babies (23.1%) had neurological signs compatible with kernicterus, including hypertonia, hypotonia, opisthotonus, high pitched cry, and seizures. All six of these babies with neurological signs had a total bilirubin level above 460 *μ*mol/L. Seventeen of the readmitted babies (65.3%) had a bilirubin level above 460 *μ*mol/L. No readmitted babies died.

Changes in haematology and blood chemistry before and after exchange transfusion are shown in Table 3. The total bilirubin and platelet counts were significantly decreased after the ET, whereas calcium and phosphate levels both significantly increased after ET (see Table 3).

### 3.2. In Hospital Babies

Most babies who underwent ET were in-hospital babies (38/64; 59.4%); 17/38 (44.7%) were very low birth weight (VLBW) infants. There were 6468 neonatal admissions during the study period; thus there were 5.8 ET per 1000 neonatal admissions. One in-hospital infant required a second ET; this was a term infant with Rh incompatibility. There were three deaths, two in VLBW babies. None of the deaths occurred during the ET. Two of the babies died of presumed sepsis at 4 and 6 days of age, respectively; both had raised C reactive protein with clinical signs of sepsis but negative cultures. The third infant was premature and died of* Escherichia coli* sepsis with perforated necrotising enterocolitis, three days after exchange transfusion.

Changes in haematology and chemistry for in-hospital babies are shown in Table 4. The total and direct bilirubin levels as well as the platelet count decreased significantly after ET. Calcium and phosphate levels both increased significantly after ET.

## 4. Discussion

Severe hyperbilirubinemia remains a problem in neonatal care in LMICS [8]. In the present study, there were 64 babies who underwent ET over a period of 4.5 years corresponding to 0.9 ET per 1000 live births. This is considerably less than that reported in Lagos, Nigeria, where 1.9% of all neonates had an ET [10]. Reasons for the lower rate of ET in the present study may reflect the fact that the hospital is in a metropolitan area, where most babies are delivered within a health facility and G6PD deficiency is much less prevalent in South Africa than in Central and West Africa [11].

It is worrying to note that six (23%) of the babies readmitted for ET had isoimmune haemolysis, suggesting that this condition was overlooked at birth. Isoimmune haemolysis is a definite risk factor for severe hyperbilirubinemia and these babies should have been closely monitored after birth. In the present study, most babies with hyperbilirubinemia were male, breastfed, and delivered vaginally, which is in keeping with other reports [12].

The present study highlights the fact that there are two populations of newborn babies who undergo ET, premature and sick babies who remain in hospital after birth and well term babies who are discharged on the day of birth. The prevention of severe hyperbilirubinemia is different for these two groups. For all babies, it is important to know the mother's blood group and to screen babies for jaundice in the first 48 hours after birth.

The fact that the majority of babies undergoing ET were “in-hospital” patients may reflect suboptimal care; however the rate of ET in hospital was relatively low at 5.8 per 1000 neonatal admissions. Reasons for inadequate monitoring of total serum bilirubin (TSB) in hospital were not evaluated in this study but may reflect inadequate staffing with high volumes of patients. Prevention of severe hyperbilirubinemia in premature and sick infants in hospital includes implementing a protocol for monitoring TSB, either using a transcutaneous bilirubin meter (TCB) or taking blood to determine TSB. House staff must be adequately trained and supervised to ensure that TSB results are obtained timeously and that babies are properly managed. Blood results may often be overlooked in busy, short staffed neonatal units, particularly after hours. It is also essential to ensure that phototherapy is effective; for example, the baby needs to be fully exposed to the light (not covered in a diaper), the luminescence of the lights must be adequate, and the lights must be the correct distance away from the baby.

The avoidable factors associated with severe hyperbilirubinemia were unknown in the majority of readmitted babies in the current study. It is, however, standard practice in the study hospital for healthy newborn babies and their mothers to be discharged within 6 hours of birth. Prevention of severe hyperbilirubinemia in babies discharged on the day of birth should focus more on factors outside the hospital setting. Prevention of severe hyperbilirubinemia includes educating expectant mothers and their families at antenatal clinic, how to detect jaundice, the correct steps to take if their baby is jaundiced, the dangers of haemolytic agents and certain traditional remedies, and the need for follow-up of babies discharged on the day of birth, especially those with risk factors for hyperbilirubinemia [7, 13]. Education of primary health care workers about the dangers, signs, and management of neonatal hyperbilirubinemia is also essential [7]. Establishing breastfeeding with proper lactation support at home after hospital discharge, especially in circumstances where most well mothers and babies are discharged on the day of birth, is also very important.

It is necessary to measure the TSB in a baby with visible jaundice. Although TCB is ideal for this purpose, these instruments are expensive and may not be freely available in LMICS [13]. “Point of care” blood testing using microsampling techniques to determine TSB may be a viable alternative [13].

The present study showed that ET was effective in decreasing the TSB. There were few complications of ET; these included a relative decrease in platelet count and increase in serum phosphate and calcium levels. The most serious complication was apnoea requiring mechanical ventilation in three babies. No babies died during the ET. The three babies who died were ill with suspected or proven sepsis. Whether the ET contributed to their demise is not clear.

### 4.1. Limitations of the Study

This was a retrospective study, so not all information could be retrieved. The avoidable factors resulting in severe hyperbilirubinemia and kernicterus were not evaluated in the current study. The study was conducted in a metropolitan area and tertiary hospital, so it is not necessarily representative of all babies with hyperbilirubinemia in South Africa. Unfortunately there is no long term follow-up data available on the babies in the present study, so the rate of neurodevelopmental problems and hearing loss from severe hyperbilirubinemia is not known.

## 5. Conclusions

Although severe neonatal hyperbilirubinemia is less common in CMJAH than in other African countries, six normal term babies were readmitted with signs of kernicterus. There is still a need for vigilant screening and correct management of babies with severe hyperbilirubinemia to prevent the occurrence of kernicterus. Monitoring of babies with the risk of isoimmune haemolysis is especially important.

## Figures and Tables

**Table 1:** Characteristics of all babies undergoing exchange transfusion.

Variable	
Male gender (frequency (%))	37/64 (57.8)
Inborn (frequency (%))	39/64 (60.9)
Caesarean section (frequency (%))	20/54 (37.0)
5-minute Apgar < 7 (frequency (%))	4/64 (6.3)
Breastfed (frequency (%))	18/51 (35)
HIV exposed (frequency (%))	8/49 (16.3)
Age at exchange (days) (mean (SD))	4.5 (2.1)
Gestational age (weeks) (mean (SD))	35.4 (4.4)
Birth weight (grams) (mean (SD))	2.29 (0.89)

**Table 2:** Comparison of readmitted and in-hospital babies who underwent exchange transfusion.

Variable	Readmitted babies (total 26)	In-hospital babies (total 38)	*p* value
Male gender (*n* (%))	17 (65.4)	20 (52.6)	0.45
Birth weight (Kg) (mean (SD))	2.7 (0.6)	2.1 (0.9)	<0.01
Gestational age (weeks) (mean (SD))	37.3 (2.4)	34.7 (4.9)	0.01
Vaginal delivery (*n* (%))	18 (69.4)	19 (50)	0.19
Signs of kernicterus (*n* (%))	6 (23.1)	NA	
Total bilirubin before ET *μ*mol/L (mean (SD))	507.4 (127.8)	394.9 (140.2)	<0.01
Breastfed (*n* (%))	20 (76.9)	16 (42.1)	0.01
Age at ET (days) (mean (SD))	4.6 (2.3)	3.3 (2.2)	0.03
Second ET (*n* (%))	2 (7.6)	1 (1.5)	0.77
Intravenous immune globulin	1 (3.6)	0	
Positive blood cultures (*n* (%))	7 (26.9)	5 (13.1)	0.28
Gram negative	2/7 (28.5%)	2/5 (40%)	
Gram positive	5/7 (71.4%)	3/5 (60%)	0.57
Cause of hyperbilirubinemia (*n* (%))			
Isoimmune haemolysis	6 (23.1)	3 (7.8)	
ABO incompatibility	2 (7.6)	1 (2.6)	
Rh incompatibility	4 (15.3)	2 (5.2)	
Cephalhaematoma	1 (3.6)	0	0.09
Red cell membranopathy	0	1 (2.6)	
Severe intraventricular haemorrhage	0	2 (5,2)	
Complications of ET (*n* (%))			
Apnoea requiring mechanical ventilation	1 (3.6)	2 (5.2)	
Glucose instability	0	2 (5.2)	
Died	0	3 (7.8)	0.18

**Table 3:** Changes in haematology and chemistry in readmitted babies before and after exchange transfusion.

Variable	Before	After	*p* value
Total bilirubin *μ*mol/L (mean (SD))	507.4 (127.8)	289.8 (59.8)	<0.001
Direct bilirubin *μ*mol/L (median (range))	34 (21.5–55.5)	22 (9.0–51.5)	0.463
Haemoglobin g/dL (mean (SD))	13.2 (2.6)	14.0 (1.8)	0.306
White cell count ×10^9^/L (median (range))	11.0 (7.1–15.4)	9.8 (5.6–12.7)	0.191
Platelets ×10^9^/L (mean (SD))	257.4 (106.0)	109.1 (79.1)	<0.001
Sodium mmol/L (median (range))	145 (139.5–148.0)	144.0 (136.0–146.5)	0.324
Urea mmol/L (mean (SD))	5.9 (4.7)	4.9 (3.8)	0.498
Creatinine *μ*mol/L (median (range))	46.0 (36.0–67.0)	55.0 (35.0–61.8)	0.726
Calcium mmol/L (median (range))	2.4 (2.3–2.5)	3.2 (2.7–3.5)	0.001
Magnesium mmol/L (median (range))	0.88 (0.76–0.95)	0.84 (0.76–0.90)	0.824
Phosphate mmol/L (mean (SD))	1.9 (0.4)	2.5 (0.4)	0.002
C reactive protein mg/mL (median (range))	4.0 (1.4–9.4)	4.0 (1.3–13.7)	0.678

**Table 4:** Changes in haematology and chemistry for in-hospital babies before and after exchange transfusion.

Variable	Before	After	*p* value
Total bilirubin *μ*mol/L (mean (SD))	394.9 (140.2)	242.5 (159.3)	<0.001
Direct bilirubin *μ*mol/L (median (range))	24 (12.5–28.5)	14.0 (10.3–14.0)	0.034
Haemoglobin g/dL (mean (SD))	14.0 (3.2)	14.3 (1.9)	0.638
White cell count ×10^9^/L (mean (SD))	11.9 (7.2)	9.8 (9.2)	0.161
Platelets ×10^9^/L (mean (SD))	225.6 (121.1)	155.9 (136.7)	0.02
Sodium mmol/L (median (range))	143.0 (140.3–146.0)	142.0 (138.0–143.0)	
Potassium mmol/L (mean (SD))	4.4 (0.8)	4.3 (0.7)	0.473
Urea mmol/L (median (range))	4.0 (2.5–5.8)	3.5 (1.8–6.8)	0.443
Creatinine *μ*mol/L (median (range))	61.5 (49.3–77.5)	60 (33.0–81.5)	0.211
Calcium mmol/L (mean (SD))	2.5 (0.3)	3.0 (0.6)	0.007
Magnesium mmol/L (mean (SD))	0.9 (0.1)	0.9 (0.1)	0.606
Phosphate mmol/L (median (range))	2.0 (1.6–2.1)	2.5 (1.5–2.9)	0.016
C reactive protein mg/mL (median (range))	2.5 (1.0–6.3)	3.1 (1.0–12.9)	0.65
